# Biogeography of Human Infectious Diseases: A Global Historical Analysis

**DOI:** 10.1371/journal.pone.0106752

**Published:** 2014-10-01

**Authors:** Elizabeth Cashdan

**Affiliations:** Department of Anthropology, University of Utah, Salt Lake City, Utah, United States of America; Curtin University, Australia

## Abstract

**Objectives:**

Human pathogen richness and prevalence vary widely across the globe, yet we know little about whether global patterns found in other taxa also predict diversity in this important group of organisms. This study (a) assesses the relative importance of temperature, precipitation, habitat diversity, and population density on the global distributions of human pathogens and (b) evaluates the species-area predictions of island biogeography for human pathogen distributions on oceanic islands.

**Methods:**

Historical data were used in order to minimize the influence of differential access to modern health care on pathogen prevalence. The database includes coded data (pathogen, environmental and cultural) for a worldwide sample of 186 non-industrial cultures, including 37 on islands. Prevalence levels for 10 pathogens were combined into a pathogen prevalence index, and OLS regression was used to model the environmental determinants of the prevalence index and number of pathogens.

**Results:**

Pathogens (number and prevalence index) showed the expected latitudinal gradient, but predictors varied by latitude. Pathogens increased with temperature in high-latitude zones, while mean annual precipitation was a more important predictor in low-latitude zones. Other environmental factors associated with more pathogens included seasonal dry extremes, frost-free climates, and human population density outside the tropics. Islands showed the expected species-area relationship for all but the smallest islands, and the relationship was not mediated by habitat diversity. Although geographic distributions of free-living and parasitic taxa typically have different determinants, these data show that variables that influence the distribution of free-living organisms also shape the global distribution of human pathogens. Understanding the cause of these distributions is potentially important, since geographical variation in human pathogens has an important influence on global disparities in human welfare.

## Introduction

Geographic variation in infectious disease has played a major role in determining history's political and demographic winners and losers [1], [2], and remains a significant factor shaping differential welfare across the world today. We know a great deal about the ecological conditions that influence the distribution of particular pathogens in particular parts of the world, but there have been comparatively few analyses of global pathogen distributions and their determinants. On the other hand, theory in geographical ecology has addressed global patterning in species distributions across a wide range of taxa. The aim of this paper is to evaluate some of those arguments in the context of human pathogens, by assessing the relative influence of environmental variables that have been found to shape species diversity in other taxa. Among the factors considered are climate (temperature and precipitation), island size and isolation, and human factors that enhance disease transmission (population density, sedentism, and roads).

The dataset is unusual in being historical and in taking as units of observation the local pathogen and environmental conditions prevailing at 186 mostly small-scale non-industrial societies around the globe (the Standard Cross-Cultural Sample, or SCCS). The data are specific to these locations, which are for the most part not near major population centers and transportation hubs. While the use of historical pathogen data poses obvious limitations in accuracy and precision, it has the potential to give a clearer picture of the role of the physical environment, since influential moderators (global travel, modern medicine and public health) played a smaller role than they do today. The more that global disease patterns rest on differential access to vaccines and antibiotics, good sanitation, and clean water, the more difficult it becomes to isolate the effect of climate and other biogeographical variables in a global analysis. The dataset also has the advantage, when compared to national data such as GIDEON, of being spatially focused and on a consistent scale. Finally, a number of relevant cultural variables have been coded for the SCCS, including several that are likely to affect pathogen abundance and diversity. The present study, therefore, complements global biogeographical pathogen analyses that have used modern datasets [3], [4]. The analysis considers effects of latitude, climate, island size and area, and population density and mobility.

Species richness is greater at lower latitudes across a wide range of taxa, and there are reasons why we might expect this to hold for human pathogens also: parasite richness is strongly correlated with host species richness in area-based studies [5], and we know that host species are typically more diverse near the equator. Furthermore, parasite-associated host mortality is greater at lower latitudes [6]. However, data on latitudinal gradients in parasites are conflicting. A recent meta-analysis across a wide range of host species found no overall relationship between latitude and parasite species richness per host species [7], and among carnivores the opposite pattern was found, with more parasite diversity on hosts living far from the equator [8]. It is likely that the influence of latitude in such studies is obscured by differences among host species that affect parasite richness (host body size, density, geographical range), a problem that would be avoided by studying pathogen diversity on a globally-distributed host such as Homo sapiens [7], [9]. Global studies of species richness in human pathogens have found such latitudinal gradients [3], [4].

The reason for latitudinal gradients in species richness remains a subject of debate [10], [11]. Energy and water availability affect organism abundance because they are central to metabolism, but it is less clear why more energy or water would lead to greater number of species; it is likely that there are several mechanisms, and that they vary by taxa [12]. Empirical studies have shown that temperature (used as a proxy for energy availability) is often correlated with species richness, but other studies have shown similar patterning with precipitation and habitat diversity. This study assesses the relative importance of these variables as predictors of historical pathogen number and prevalence. In addition to developing a global model, the study tests the hypothesis that temperature is more important in areas where it is limiting (i.e., areas far from the equator), while water [13] and habitat diversity [14] are more important in areas of energy abundance.

Species richness on islands is also shaped by island size and isolation. The MacArthur & Wilson [15] equilibrium model of island biogeography explained this relationship as a consequence of immigration and extinction rates: smaller islands have fewer species due to higher extinction rates and fewer habitats, and more isolated islands have fewer species due to lower colonization rates. Larger islands also attract more immigrants (target effect) and less isolated islands receive repeated immigration and so are less vulnerable to extinction (rescue effect). While the assumption of equilibrium is problematic and new dynamic theories have been developed [16], [17], the influence of island area and isolation remain important. A separate analysis of 37 islands in the sample was therefore conducted to see whether the size and isolation of islands shape pathogen number and prevalence, and, if so, whether greater habitat diversity on larger islands could explain the relationship.

Finally, the SCCS also allows us to include in the models aspects of human demography and culture likely to affect pathogen growth and transmission. Host population density is a strong predictor of parasite species richness across a wide range of host taxa [7], including non-human primates [18], and the same is likely to be the case for humans. Skeletal and other evidence suggests that the neolithic transition to settled farming and husbandry was often accompanied by an increase in infectious disease; proposed reasons include the larger pool of susceptible hosts and wider contacts arising from larger, denser, and more permanent settlements, as well as exposure to new zoonoses and vectors associated with food production [19]–[21]. Similar factors are likely to lead to variation in pathogen exposure among the nonindustrial societies of the SCCS. These factors are evaluated here by modeling the effects of population density, sedentism, and road quality.

The analyses begin by looking at the environmental variables that affect pathogen diversity and prevalence globally. The sample is then divided into tropical and non-tropical regions, and the relative importance of these environmental factors in the two regions is compared. Finally, a set of analyses was performed on the island locations only, in order to evaluate predictions about the effects of island area, isolation, and habitat diversity.

## Methods

### Data sources

**Figure 1:**
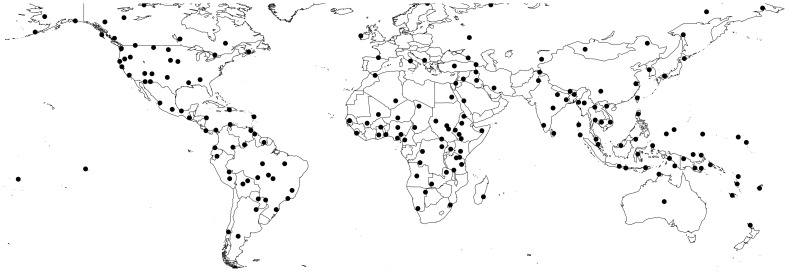
Standard cross-cultural sample locations.

The analyses use data from the Standard cross-cultural sample (SCCS) of 186 non-industrial cultures (see Figure 1). Each SCCS society is pinpointed to the time and location of a key ethnographic description [22], with most dating to the early part of the twentieth century (interquartile range 1880–1939). Many sociocultural and environmental variables have been coded for this sample, with the open access electronic journal World Cultures (http://www.worldcultures.org) functioning as a repository. This paper uses both existing coded data for the SCCS and a newly-developed set of SCCS pathogen codes.

The new pathogen codes are described briefly below and in Cashdan & Steele [23], and in more detail in the supplementary materials. Datafile S1 includes the code and information to guide its use and interpretation, while Datafile S2 contains the coded data.

#### Pathogen data

The pathogen data for the new codes were derived from historical sources, chiefly global maps published in the mid-twentieth century. The codes reflect the prevalence levels of 8 pathogens: malaria, dengue, filariae, typhus, trypanosomes, leishmanias, schistosomes, and plague. Most of these pathogens include several related species, due to limitations of the source material. Prevalence levels were taken primarily from isolines on the epidemiological maps, and coded as 1  =  absent, 2  =  rare, 3  =  sporadic or moderate prevalence, and 4  =  epidemic or high prevalence. The prevalence levels of the different pathogens were combined, as described below, to form a pathogen prevalence index.

The coding procedure followed that used by Murray & Schaller [24] in their historical cross-national pathogen codes, but was made specific to local conditions by recording, for each of the 8 pathogens, the highest pathogen level (1–4) within a 100 km radius of each SCCS society. The main sources were the three volume series of maps in Rodenwaldt & Bader [25] and the maps and data in Simmons et al. [26], supplemented by data in Faust & Russell [27]. Low [28], [29] developed a 7-pathogen index for the SCCS using different historical sources. The two codes are highly correlated, but Low's includes two pathogens (leprosy and spirochetes) not in the Cashdan-Steele dataset. A combined index was therefore created by converting Low's three-point scale for leprosy and spirochetes and the Cashdan-Steele four-point scale for the other eight pathogens to z-scores, and using the mean of the 10 z-scores as an index of pathogen prevalence (see Datafile S1). A high score on the index, therefore, indicates both more types of pathogens and more severe exposure. In order to get a measure that more closely reflects species richness, a second index was created in which pathogens were dichotomized as either present or absent. The score here is the number of pathogens out of a total possible of 10. All analyses were done with both the pathogen prevalence index and with number of pathogens.

Because of limitations in the source material, both codes are biased toward pathogens that are transmitted through arthropod and other vectors. A few of these also have non-human hosts. The prevalence of such diseases is likely to be strongly shaped by the geographic distribution of the vectors that transmit them and the species that host them. This bias has the disadvantage that a number of important diseases (e.g., measles and cholera) are omitted. It also has the advantage that the geographic patterning of this sample of diseases will be less affected by international travel and by socioeconomic and public health measures than are diseases spread via droplet and oral-fecal transmission.

Another limitation is that the historical data do not contain information on variation in sampling effort, and less was known about pathogens in remote areas like tropical Africa than in more economically developed parts of the world. Since the sources nonetheless indicate more pathogens in these tropical regions, particularly in central Africa, the effect of this bias is likely to be conservative. Sampling bias is likely to be most problematic in studying the influence of island area, since pathogens on very small islands might have been estimated from better-known larger islands in the vicinity. The implications of this potential bias is discussed in the results section on island analyses.

#### Environmental and island data

Energy measures included in this study were mean annual temperature, number of frost-free months, and within-year measures of temperature extremes [30]. Water availability was measured by yearly mean precipitation over a 20-year period [31], and within-year measures of wet and dry extremes, including lowest precipitation in dryest month and highest in wettest month [30]. All data were taken from weather station records closest in time and place to the focus of each SCCS society. Habitat diversity was coded as the number of vegetation types within a radius of 100 through 250 miles [31], [32], based on world maps published in the 1960s [33]. Many sociocultural factors affect pathogen spread, directly or indirectly, and three are used in these analyses: population density [34], road quality [35], and sedentism [34]. These are ordinal variables, as described below. The environmental and cultural data analyzed here come from the 2003 World Cultures 14(1) data disks, although the original published sources were consulted for full variable definitions and coding procedures.

Island area and various measures of isolation were obtained from the UNEP (United Nations Environment Programme) Island Directory at http://islands.unep.ch, supplemented in a few cases by other sources. A few islands were so small that the 100 km radius used to calculate pathogens extended beyond the island border. In these cases, if there was another island within that radius, the area of that island was added to the focal island.

### Analysis

There are two parts to the analysis. The first uses the full sample of 186 locations to build a global model of significant environmental predictors of pathogen prevalence (using the prevalence index) and richness (using number of pathogens). The global model was built incrementally, beginning with a model of physical environmental variables (island vs. mainland, temperature, and precipitation) followed by a separate model of three related cultural environmental variables (density, sedentism, road quality). The significant predictors from the two models were then combined into a single global model. In each case, analysis began with single-factor regressions followed by multivariate models, and variables that were individually significant but did not contribute independently to the multivariate models were dropped. The model was then applied separately to tropical and non-tropical regions, because the strength of these predictors was hypothesized to differ by latitude. Because the aim of the global model was to compare the relative effects of the different predictors, multiple regressions report standardized (beta) coefficients.

The second part of the analysis uses only the subset of 37 island locations in order to test the specific hypotheses that pathogen number and prevalence are associated with island size and habitat diversity.

In conducting the regressions, island area and some climate variables were transformed with a natural log transform prior to regression in order to make relationships linear and improve residual distributions. Where necessary, a constant was added before the log transform in order to make the minimum value 1.0. Mean annual temperature was negatively skewed, so those data were also reflected about zero before the log transform and then reflected back so as to restore the original order.

The ordinal variables were handled in different ways, depending on the nature of the variable. Road quality was dichotomized into societies where only footpaths were present, originally coded 1 (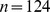), and societies with roads of varying quality, originally coded 2–4 (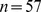). Sedentism was dichotomized into the 117 societies that maintain permanent camps (5–6 in the original scale) and the 69 that move during the year (1–4 in the original scale). Unlike road quality and sedentism, which were defined by qualitative descriptors, the 7 levels of population density correspond nonlinearly to persons per square mile: (1) less than 1 person per 5 sq. mi, (2) 1 person per sq. mi – 1 person per 5 sq. mi, (3) 1.1 – 5 persons per sq. mi, (4) 5.1–25, (5) 26–100, (6) 101–500, and (7) more than 500 persons per sq. mi. Population density was analyzed in multivariate regressions as an interval variable, although the underlying density in persons per square mile cannot be directly inferred from the data. Prevalence levels of individual pathogens were used only in bivariate correlations with individual environmental variables, using Spearman's rank order correlations.

#### Validity checks

Two additional analyses were done to validate the global model. The first was to run it separately against the two codes from which the combined index was derived. This was done both as a check on coding accuracy (since the codes used different historical sources) and as a way to evaluate how vulnerable the model was to the particular pathogens chosen (since the codes differed somewhat in the diseases coded). Another check was done to see whether the dependent and independent variables were associated only because they varied similarly across space, which would be indicated if there was spatial autocorrelation in the residuals. For each pair of points, the (squared) difference between the residuals and the actual geographic distance was calculated, to see whether the two values were correlated. This was done at various scales of distance down to 200 km. The societies in the sample are geographically dispersed (stratified both by geographic region and language group) so spatial autocorrelation at smaller scales cannot be assessed.

SAS was used for all analyses.

## Results

The first part of the analysis builds a global model using the full dataset, first by considering the physical environment, then the cultural environment, and finally both together in a single model. The sample is then divided into high and low latitude zones, to see how the relative importance of these variables differ by latitude. The final analyses are restricted to the island locations, in order to test specific predictions from island biogeography.

### Global Analyses

#### Latitudinal gradients

**Figure 2:**
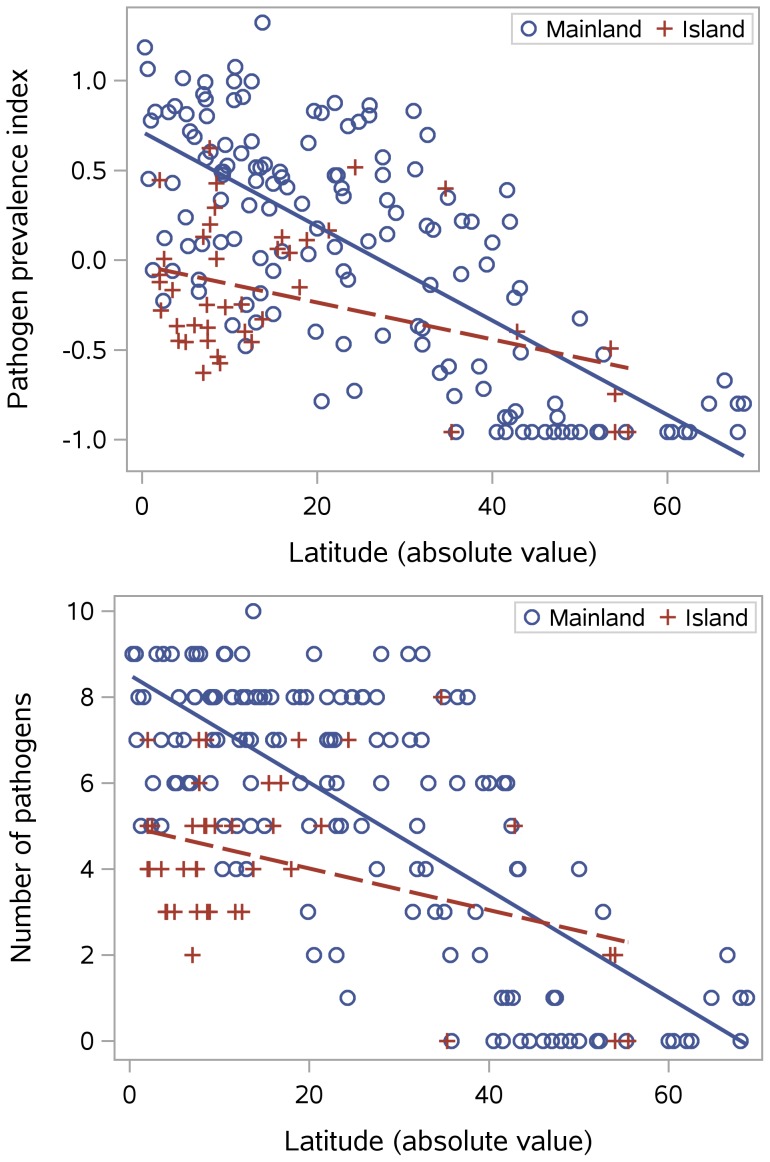
Pathogens by latitude. Separate regression lines for mainland and island locations.

The upper graph in Figure 2 shows that the pathogen prevalence index is negatively correlated with distance from the equator, particularly when island locations are excluded, and that island locations have lower pathogen scores than those on the mainland. Because the pathogen prevalence index conflates number of species and abundance, the lower graph uses an index based solely on pathogen presence or absence; it shows a similar picture, with islands having fewer pathogens than expected given their latitude. This result is consistent with the broader literature on island biogeography, which finds species richness to be reduced on islands, and will be discussed further in a later section that considers island area. First we turn to the climatic factors that might be influencing the latitudinal gradient. In this dataset, mean annual temperature and precipitation are both correlated with distance from the equator (mean annual temperature: 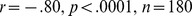; mean annual precipitation: 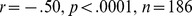), so the first question is which variable is more important in shaping pathogen distributions, and to what extent associated variables (climate extremes and variation) also play a role.

#### The physical environment (temperature, frost, precipitation)

**Figure 3:**
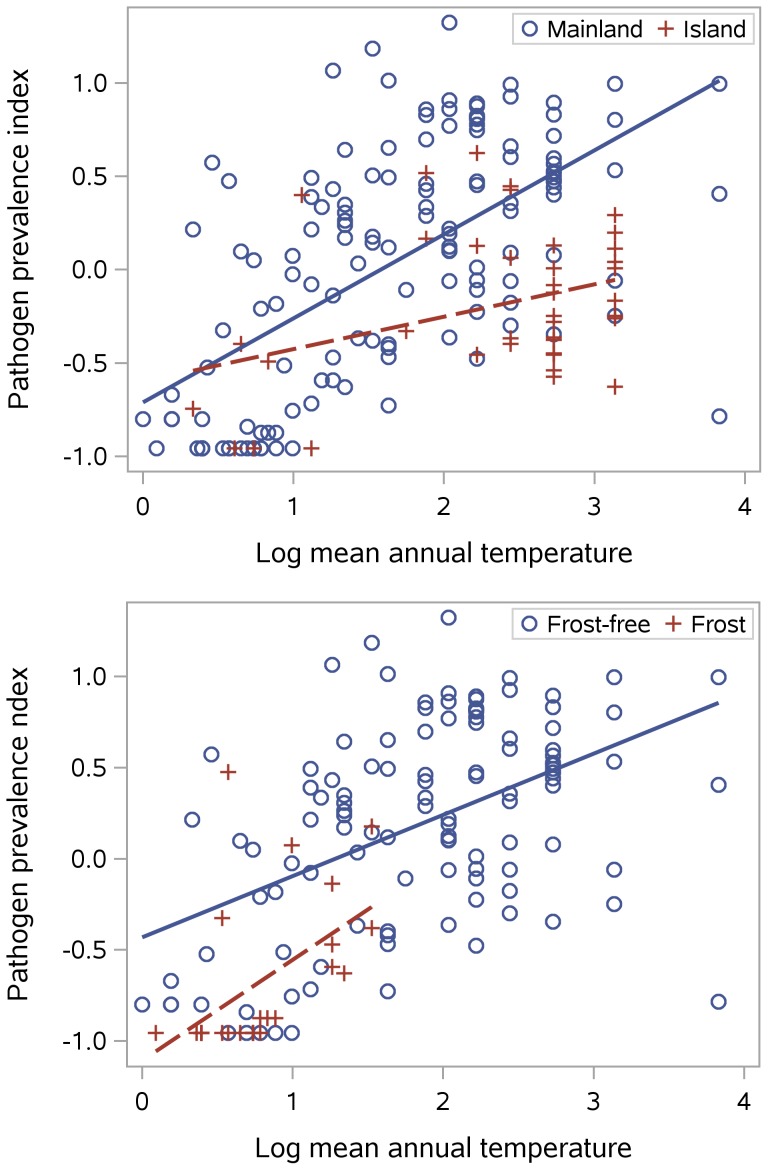
Pathogens by natural log of mean annual temperature (c). Regression lines in the upper graph are subset by mainland vs. island locations. Regression lines in the lower graph are subset by locations with frost-free climates vs. those with one or more frost months.

Figure 3 shows pathogens as a function of log mean annual temperature, subset in two ways to illustrate additional effects on the relationship. The upper graph shows that islands have lower pathogen scores than would be expected from their temperature, the same pattern seen with latitude. The lower graph, which excludes islands, shows that a year-round frost-free climate predisposes to more pathogens than would be expected from the climate's average temperature. Other measures of within-year temperature extremes were also analyzed, but were too highly correlated with mean annual temperature to be included in regressions. Temperature, frost, and islands have independent effects when included together in a multiple regression model: log mean annual temperature, frost months (dummy coded as some vs. none), and islands (dummy coded as island vs. mainland) together explain 39% of the variance in the pathogen prevalence index and 40% of the variance in number of pathogens, with the pathogen prevalence index being higher on the mainland (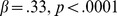), in areas with high mean annual temperature (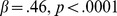), and in frost-free climates (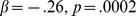).

**Figure 4:**
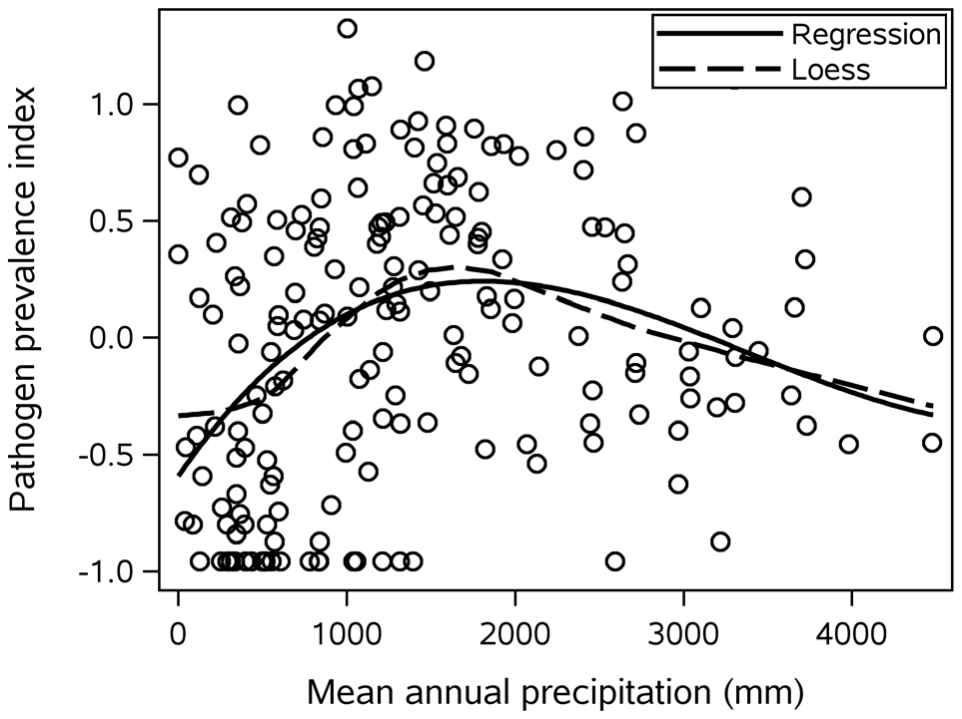
Pathogen prevalence index by mean annual precipitation. The regression line is a third-order polynomial.

**Figure 5:**
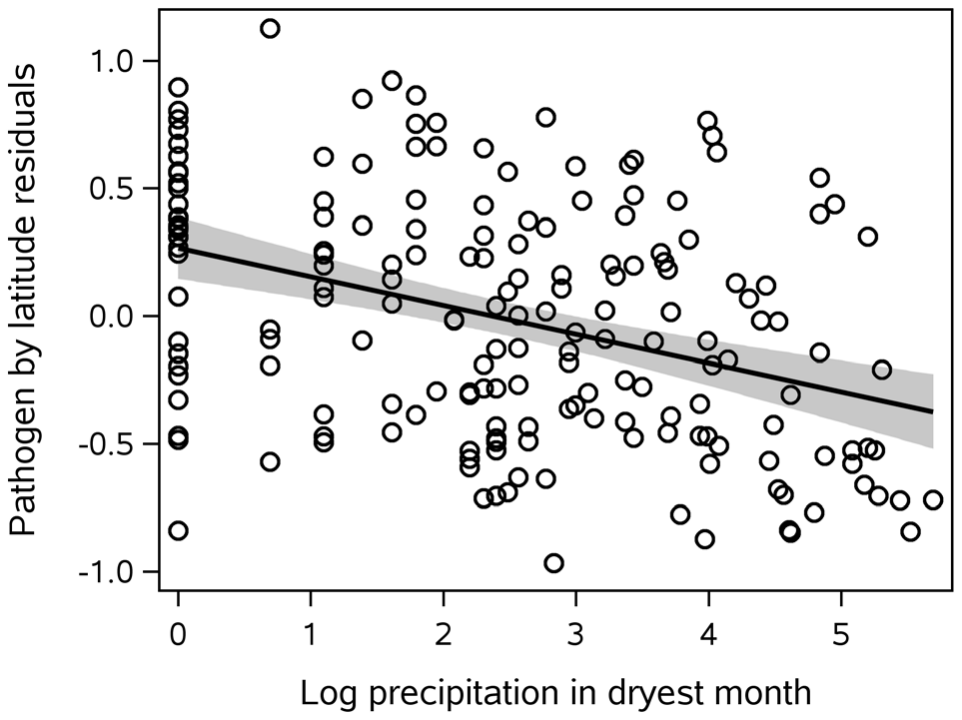
Pathogens by seasonal dry extremes, controlling for latitude. Dry extremes are measured by the lowest precipitation in the dryest month, logged. Regression with 95% confidence limits.

Precipitation shows a more complicated relationship to pathogens, because two variables have independent effects: (a) mean annual precipitation and (b) the amount of precipitation in the dryest part of the year. As will be shown below, these two precipitation variables affect different kinds of pathogens. Mean annual precipitation showed a modest (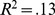) curvilinear relationship with pathogens best approximated with a third-order polynomial (see Figure 4; one influential precipitation value was removed from this graph and from the analyses). Extreme dryness during part of the year (measured as log lowest precipitation during driest month) also increases the pathogen prevalence index (see Figure 5). Adding mean annual precipitation and seasonal dry extremes to the previous model increases the variance explained to 50% for both the pathogen prevalence index and number of pathogens.

This final climate model, showing the effects of the physical environmental variables (island vs mainland, temperature, frost, mean rainfall and seasonal dry extremes) has an adjusted 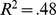, 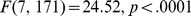 for the pathogen prevalence index and adjusted 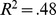, 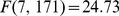 for number of pathogens.

It has been suggested [36] that greater climate variation leads to lower diversity because organisms in such climates have evolved to be generalists, broadly tolerant of a wide range of climates. Guernier et al. [4] found the opposite to be the case for six groups of human pathogens: greater seasonal range in precipitation was associated with greater species diversity. In the present analysis, also, greater precipitation range (measured as maximum precipitation in wettest month minus lowest precipitation in dryest month) was associated with a higher pathogen prevalence index. However, dry extremes seem to be driving this relationship; precipitation range was not a significant predictor when the other climate variables are included in the model, while lowest precipitation in dryest month (one componenent of precipitation range) remains significant.

The effects of temperature, mean precipitation, and dryness differ for the different pathogens, and the patterning appears to reflect the ecology of the vector more than the type of pathogen. The mosquito-borne pathogens are a variable lot, including malaria (protozoans), dengue (virus), and filariae (nematodes), but all were worse in hot wet climates. Typhus (rickettsia) leishmanias (protozoans), and schistosomes (flukes) were all worse in areas with dry months, perhaps because of greater aggregation of vectors and hosts during drought. Bivariate correlations between the various pathogen groups and environmental predictors are summarized in Table 1.

**Table 1:** Pathogen-specific correlations.

	Mean annualTemperature	Yrly Precip(linear)	Yrly Precip(polynomial)	PrecipitationDryest month	Number ofFrost months	PopulationDensity
Malaria	.53***	.42***	.41	−.07	−.41***	.36***
Dengue	.56***	.48***	.43	.06	−.38***	.48***
Filaria	.53***	.42***	.41	−.02	−.39***	.46***
Typhus	−.07	−.01	.22	−.39***	−.16*	.35***
Trypanosomes	.06	.18*	.20	−.03	−.12	.05
Leishmanias	.21**	.08	.14	−.22**	−.33***	.11
Schistosomes	.26***	.17*	.19	−.24***	−.24**	.18*
Plague	.07	.27**	.17	.01	−.12	.19*
Spirochetes	.35***	.26**	.20	−.13	−.35***	.38***
Leprosy	.38***	.35***	.23	−.19*	−**.31** ***	.32***

Pathogen prevalence level by predictors in the final model of Table 2. Spearman's rank-order correlations were used for all variables other than annual precipitation. Two correlations are given for mean annual precipitation (“Yrly Precip”): the polynomial model uses the full sample, but without significance values, which are probably unreliable for the individual pathogen scores. The linear model is limited to the 78% of the sample where pathogens increase with precipitation (i.e., up to 2000 mm). Other variables were transformed as indicated in the text, except that frost was not dichotomized. Sample sizes are 180 for log temperature, log precipitation in dryest month, and number of frost months; sample sizes for the polynomial and linear correlations with mean annual precipitation are 185 and 143 respectively.

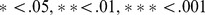

#### The cultural environment (population density, mobility, roads)

Pathogen distributions are affected by cultural as well as physical environmental factors. This section examines the effect of three cultural variables (population density, residential mobility, and roads) on pathogen distributions.

Better roads can be expected to broaden the geographic reach of pathogens by facilitating the movement of people, and of insect vectors transported inadvertently in the goods they carry. The mean pathogen prevalence index was higher in societies with roads (.29) as opposed to footpaths (−.14), 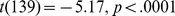. Pathogens were also higher in more sedentary groups: societies with permanent camps had a mean pathogen prevalence index of .22 as compared with −.37 for more mobile groups (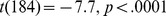). Population density was also, as expected, positively correlated with the pathogen prevalence index: 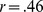 (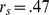), 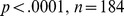.

**Figure 6:**
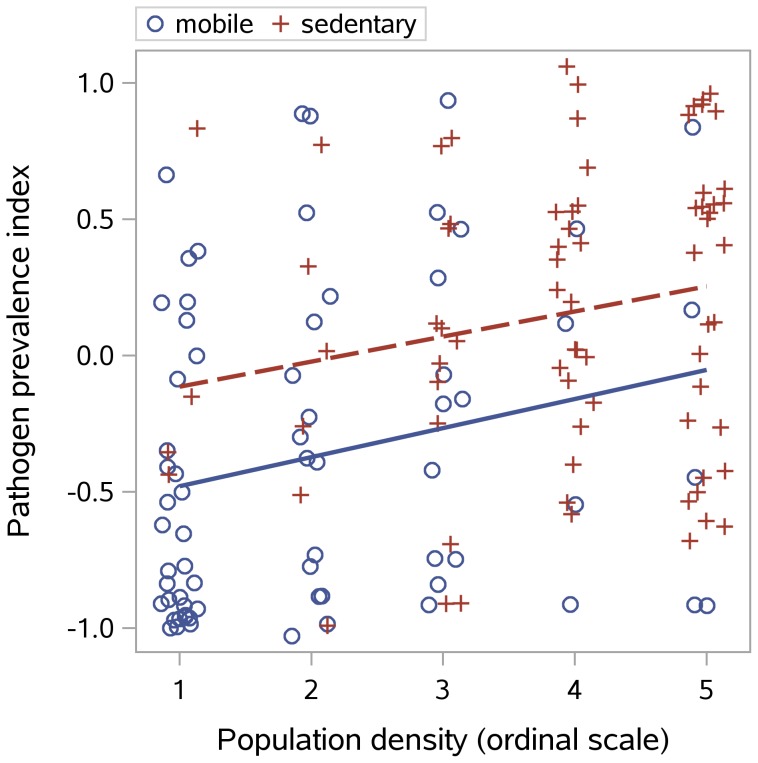
Pathogen prevalence index by density and residential mobility. Points have been jittered to avoid overlap. Population density is a nonlinear ordinal scale based on persons per sq. mi; see the methods section for density and mobility codes.

These variables are correlated, since all are associated with greater social complexity. Figure 6 shows that increases in population density are accompanied by a trend toward increased sedentism, although mobile populations have lower pathogens at the same degree of density. A check for collinearity supports keeping both sedentism and density in the model (variance inflation factor  =  1.84), although with these variables in the model, road quality is no longer a significant predictor. The cultural environmental model, with just density and sedentism, explains 26% of the variance in the pathogen prevalence index and number of pathogens. For the pathogen prevalence index, the standardized coefficients were 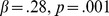 for density, 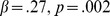 for sedentism.

Examination of outliers in the climate analysis underscores the importance of considering the cultural as well as physical environment. For example, there was a highly influential point in the temperature and frost model. This point represents the Teda, a nomadic group in Chad with an unusually low pathogen score, given their local temperature and rainfall. None of the physical environmental factors in the dataset explain the discrepancy adequately, but their comparatively low pathogens are consistent with their very low density and high mobility at the time and place of their SCCS ethnographic description.

#### A combined global model (physical and cultural environmental variables)

The effects of the physical and cultural environment on disease are not independent, and so the final global analysis considers the variables in a single model. In a combined model with the physical environmental variables, residential mobility is no longer a significant predictor and is dropped from the final model. However, the earlier result suggests that the effect of density on pathogens in this model may be due both to its direct effects and to indirect effects resulting from associated decreased mobility.

Taken together, the results indicate that there are more pathogens and pathogen types on the mainland than on islands, and that pathogens increase with mean temperature, population density, and a frost-free climate. The relationship with precipitation is more complex, peaking at intermediate levels of mean annual precipitation but also increasing in seasonally dry climates. The combined model explains 58% of the variance in both number of pathogens and the pathogen prevalence index. The regression statistics of this model are in Table 2.

**Table 2:** Final regression model for predictors of the pathogen prevalence index and number of pathogens.

	Prevalence Index		Number of Pathogens	
Variable	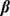	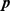	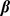	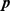
Temperature	0.30	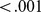	0.31	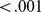
Frost months	−0.19	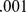	−0.20	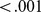
Island	0.31	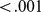	0.31	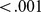
Driest month	−0.18	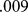	−0.19	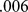
Pop Density	0.31	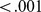	0.30	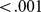
Precipitation (P)	1.78	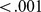	1.62	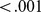
P 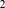	−2.73	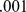	−2.45	
P 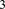	1.15	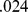	1.03	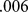
	F(8,168) = 29.57		F(8,168) = 29.23	
	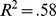		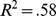	

Variable definitions:

Temperature: Log mean annual temperature (c).

Frost months: 1 = presence 0 = absence.

Island: 1 = mainland 0 = island.

Driest month: Log lowest precipitation in driest month (mm).

Pop Density: Population density (ordinal scale, 1–7).

Precipitation: Mean annual precipitation (mm).

#### Validity checks

As a check, the final model was run against each of the two databases from which the combined pathogen score was derived. Low [28], [29] coded data on 7 pathogens using different historical sources, only two of which were used in the combined index. Using Low's 7-pathogen index as the dependent variable with this model produces an identical 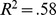, although the coefficient for density was smaller and that for temperature was larger. The greater influence of temperature using Low's data is probably because typhus and plague, which are unrelated to temperature in Table 1, were not included in that dataset. The other coefficients were similar to those of the combined index. A similar summation of the 8 pathogens in our new codes yields an 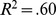 with coefficients very similar to those of the combined index.

Another check was done to see whether the relationship between the independent and dependent variables in this model was due to spatial autocorrelation (e.g., whether independent and dependent variables were associated only because they vary similarly across space). Where this is the case, there will be spatial autocorrelation in the residuals. In order to evaluate this, the squared difference between the residuals of each pair of points was plotted against their great circle geographic distance, using both the full sample and subsets at increasingly smaller scales (points less than 3000, 1000, 500, and 200 km apart). Visual inspection indicated that the relationship was flat (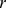 averaged 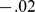) at all scales of distance, none were statistically significant, and there were no trends with distance over this range.

### Differences between high and low latitude regions

The model above is the best fit for global pathogen distributions, but recent literature suggests that more specific models may be appropriate at high and low latitudes. Energy availability appears to have a greater effect on species richness farther from the equator, whereas water [13] has been proposed as more important where energy is abundant. Habitat diversity [14] may also be more important at low latitudes. The sample was divided into tropical (low latitude) and non-tropical (high latitude) zones, and the results supported these expectations. Bivariate correlations by latitude zone are shown in Table 3.

**Table 3:** Bivariate correlations between independent variables and pathogen number and prevalence index, by latitude zone.

	Low latitudes		High latitudes	
	Number	Prevalence	Number	Prevalence
Mean temperature	−.20*	−.18	.59***	.61***
Mean precipitation	.44***	.40**	.30	.35
Low precip dryest month	−.33***	−**.31** **	−.33**	−.31**
Population density	.14	.17	.67***	.69***
Habitat diversity	.36***	.24***	.05	−.01

Note. Table shows Spearman's rank order correlation coefficients except for mean annual precipitation, where the correlation is based on the adjusted 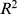 of a third-degree polynomial regression (no significance values are given for high latitudes because of poor fit diagnostics). Sample sizes for low/high latitudes: temperature 107/73, precipitation 111/75, precipitation dryest month 107/73, habitat diversity 97/75, density 109/75. Habitat diversity calculated at 150 miles radius; the correlation was slightly less at 100 miles.

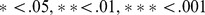

#### High latitudes

As expected, temperature was a significant predictor only at high latitudes. In this region, the relationship was strongly linear (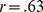 for number of pathogens, 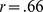 for the pathogen prevalence index). An unanticipated result was that the same is true for population density: it is a strong predictor of pathogens at high latitudes only. A multivariate model using only those two variables (log mean annual temperature and population density) explains 56% of the variance in the pathogen prevalence index and 61% in pathogen number at high latitudes. No other variables add significantly when those are in the model.

#### Low latitudes

The pattern in the tropical locations, in contrast, is shaped more by precipitation than by temperature. The relationship between precipitation and pathogens in the tropics is similar in shape to that shown in Figure 4 for the full sample, but the relationship is much tighter, the peak is at somewhat lower precipitation, and the pathogen decline at higher precipitation is more apparent. The best multivariate model of the pathogen prevalence index in the tropics includes mean annual precipitation as a third-degree polynomial together with population density (notwithstanding its weak bivariate relationship) and the dummy-coded island vs. continent (most of the islands in the sample are in the tropics). This model explains 44% of the variance in the pathogen prevalence index and 50% in pathogen number.

Table 3 shows that habitat diversity (measured as number of vegetation zones in a given radius) is also a factor in shaping pathogen diversity in the tropics. Habitat diversity remains significant when added to the other variables (precipitation, islands, density) in the tropical model. However, the overall 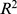 is reduced, perhaps because of reduced sample size when that variable is included. Habitat diversity, alone among the variables considered in this study, is a stronger predictor of number of pathogens than it is of the prevalence index. Habitat diversity presumably facilitates pathogen species richness via niche differentiation, whereas temperature, precipitation and population density also have direct effects on pathogen prevalence by enhancing pathogen growth and transmission.

### The island model

**Figure 7:**
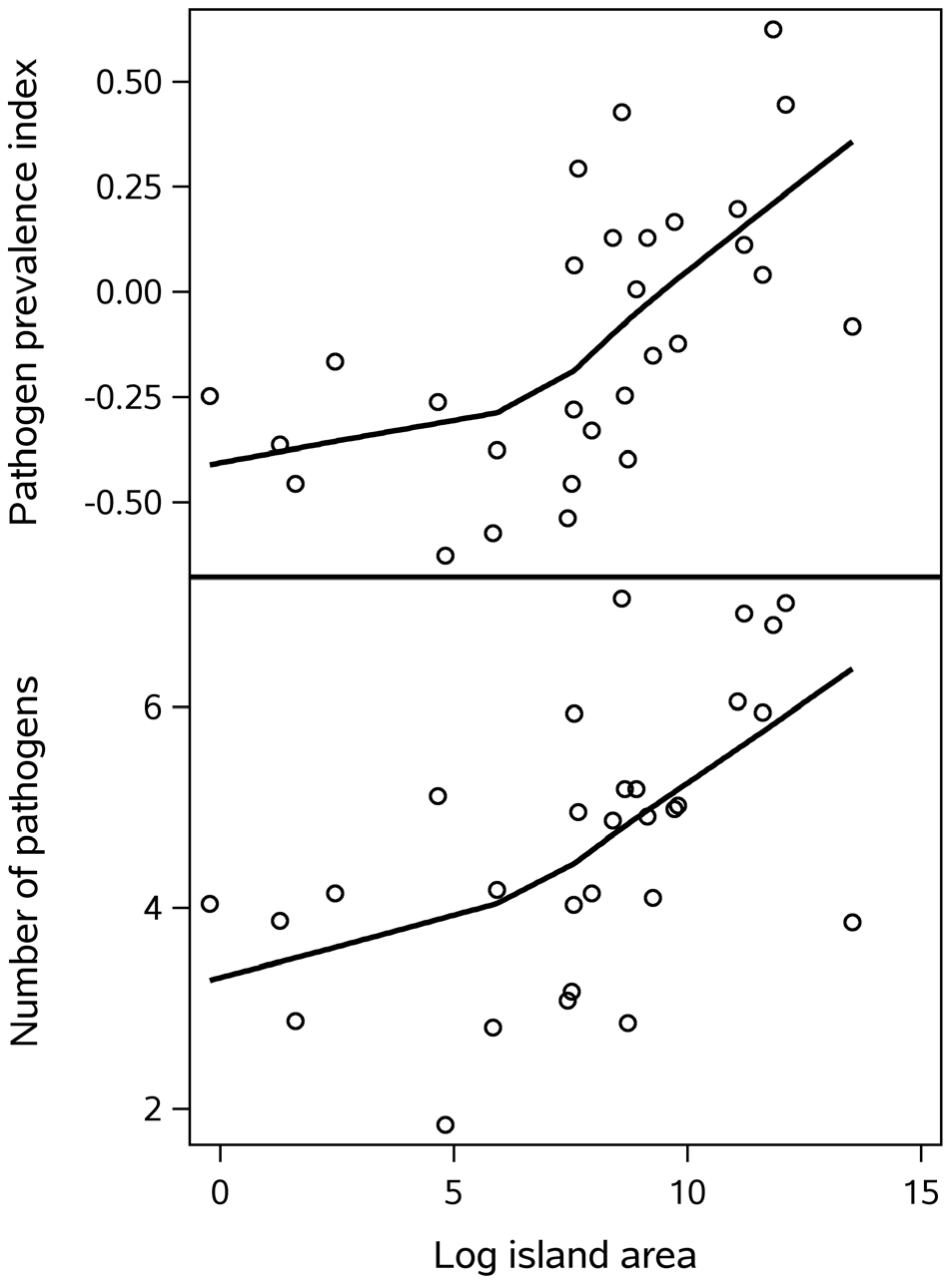
Pathogens by Island Area (tropical islands only), with loess curves.

We now turn to the subset of the sample consisting of island locations, in order to test the prediction from classical island biogeography that small islands will have fewer species than large islands. The analysis is based on 37 islands (New Guinea was excluded because it is home to four societies in the sample). The prediction is supported: controlling for distance from the equator, the partial correlation of log island area with pathogens is 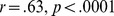 for the pathogen prevalence index and 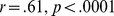 for number of pathogens. Figure 7 shows the relationship for islands in the tropics; the non-tropical islands are included in the statistics but are not shown in the figure because they span a wide latitudinal range.

As Figure 7 indicates, the linear relationship breaks down for the smallest islands. This is often the case in small islands, where the effect of area on species richness is overshadowed by stochastic factors [37], [38]. In such cases, species richness typically plateaus at the lowest level, which may not be the case in these data. The leveling off with small islands could be an artifact of the poor resolution of historical pathogen data, reflecting extrapolation from better-known larger islands to poorly-sampled small islands nearby. A regression without the four smallest islands probably presents a more accurate picture of the relationship between pathogens and island area; in this model the largest island (Borneo) is also best removed as it is a highly influential point. Within this intermediate range of values, the relationship is linear with the log of island area, and the regression of log area and latitude on pathogen number provides a better fit: 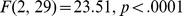, 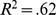. The unstandardized coefficient for log island area on number of kinds of pathogens is 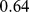.

Theory predicts that pathogens will also decrease with distance from the mainland. The relationship is weak in this dataset, and its independent effect is hard to evaluate since the smallest islands are also farthest from the mainland. Getting a good measure of isolation is difficult, since it involves not just distance to the nearest continent but to nearby islands that could be links to sources of greater diversity. Various distance measures were used to try to capture this, but none showed more than a weak correlation with pathogens, or remained significant when island area was also included in the model. However, this could reflect measurement difficulties rather than relative importance. It is also possible that some of the area effect reflects the greater isolation of many of the smaller islands.

## Discussion and Conclusions

### Summary

Many of the variables that influence the distribution of free-living taxa also predict the number and prevalence index of human pathogens in this dataset. Pathogens increased with mean annual temperature and, controlling for mean temperature, in climates that remained free of frost throughout the year. The effect of temperature was highly significant, but only outside the tropics. Within the tropics, mean annual precipitation was a more important predictor, and was associated most strongly with mosquito-borne diseases (malaria, dengue, filariasis). Extreme seasonal dryness was also associated with more pathogens, especially typhus, leishmaniasis, and schistosomiasis. Finally, pathogens were worse in areas with high population density. Pathogens were also more numerous on the mainland than on islands, and on large as opposed to small islands. Most of the predictions derived from species diversity patterns in other taxa were supported, and are discussed in turn below, beginning with the island results.

### Island biogeography

The classic model of island biogeography [15] predicts that there will be fewer species on islands that are small and isolated. The model uses simplifying assumptions [16], and the assumption of equilibrium is particularly problematic when studying the distribution of human infectious diseases. Nonetheless, and notwithstanding the heterogeneity of the 37 islands in this dataset, the classic predictions of island biogeography were upheld: islands have fewer kinds of pathogens than expected given their climate, and fewer in smaller than in larger islands. Island isolation did not have an independent effect, but may have had an indirect influence since many of the smaller islands in this dataset were also more isolated. Smaller islands also offer fewer types of habitat, but this appears not to be driving variation in pathogen richness in this dataset: although habitat diversity and area of islands were correlated, only island area had any relationship to pathogens.

The lower pathogen load on islands is consistent with Curtin's [39] meticulous accounting of historical troop mortality, which found that early in the 19th century some Pacific islands had a far lower pathogen load than would be expected by their climate, particularly Tahiti “which gave French troops a 100% mortality improvement over France” in the 1840s and continued to give benefits into the early 20th century ([39], p. 12). A similar pattern existed for Hawaii and New Zealand. (This ecological protection later made the islanders vulnerable to novel European diseases, which caused huge mortality throughout the Pacific).

Most of the pathogens in this dataset are vector-borne, and this is likely to enhance the influence of island area and isolation since two species, pathogen and vector, need to be present at the same time [40]. Some pathogens also require a minimum size of host population in order to remain endemic. This triple challenge can be expected to amplify the effect of isolation and extinction on pathogenic species on small islands, and among small isolated populations generally [41].

## Latitudinal gradients

Both the pathogen prevalence index and number of pathogens show a strong latitudinal gradient in these data, with more pathogens closer to the equator. This could reflect vector ecology, since malaria (protozoans), dengue (virus) and filariae (nematodes) are taxonomically diverse, yet all are transmitted by mosquitos and all are most prevalent in hot wet climates where mosquitos are abundant. Typhus, leishmanias, and schistosomes, on the other hand, are transmitted by other vectors and intermediates (lice and fleas, sandflies, and freshwater snails, respectively) and had different environmental correlates. The latitudinal gradient in human pathogens may also reflect the ecology of alternate hosts. Rodents, an important reservoir for many human diseases, host more viral parasites at lower latitudes, perhaps because of latitudinal gradients in their own viral vectors [42]. This complexity may contribute to the inconsistent findings regarding latitudinal gradients in parasite species richness across host taxa [7], [9].

## Environmental predictors

Although a latitudinal gradient in species richness exists across a wide range of free-living taxa, the cause of this pattern remains a topic of debate [10], [11] and its relevance to parasitic taxa remains unclear. A number of mediators have been discussed in the literature; those discussed below include temperature (often used as a proxy for energy availability), frost, and precipitation.

Pathogen prevalence levels increased with temperature in 7 of the 10 pathogen groups, including the mosquito-borne pathogens, which are known to be highly temperature sensitive [43]. Although growth and survival of insect vectors, hence pathogen transmission, decline in extreme heat [44], the relationship with log temperature was linear in these data. There were also more kinds of pathogens as temperature increased. Locations with a year-round frost-free climate had more pathogens than would be expected from their mean annual temperature, probably by enabling pathogens and their vectors to overwinter.

Precipitation had a more complex relationship with the pathogen prevalence index, because mean yearly precipitation and extreme seasonal dryness had different effects and affected different pathogens. Mean annual precipitation had a curvilinear relationship with both the prevalence index and pathogen number, peaking at intermediate values and declining in very high precipitation areas. The association between pathogens and mean annual precipitation was especially strong in the tropics and for mosquito-borne pathogens; perhaps the decline in very wet areas is associated with mosquito larvae being washed out due to heavy rains and flooding.

Areas with little or no precipitation during the dryest part of the year (lowest precipitation in dryest month) also had more pathogens. Seasonal dryness affected a different group of pathogens than did mean temperature and precipitation. The greatest effect was on typhus. Typhus is transmitted by fleas and lice and can become worse in crowded conditions with poor sanitation, and when drought causes rodents (and the fleas they carry) to move near human habitation in search of water. An analysis of tree-rings in pre-industrial Central Mexico found that a significant drought occurred during the first year of all 22 large typhus outbreaks studied [45]. A similar effect via aggregation of people and the sand fly vector during dry periods has been associated with temporal changes in leishmaniasis in Brazil [46].

Guernier et al. [4] found precipitation range to be the single best predictor of species richness across six categories of human pathogens. In the present dataset, precipation range (highest precipitation in wettest month minus lowest precipitation in dryest month) was also associated with significantly higher pathogen number, and with the prevalence index. However, range was not a significant predictor when other climate variables were included in the model, while seasonal dryness (one component of range) remained significant. In these data, therefore, the more influential aspect of precipitation range on pathogen distributions appears to be seasonal dryness.

## Differences at low and high latitude

The relative importance of temperature and water as predictors of species richness has been shown to vary with latitude, with temperature being more important at high latitudes and availability of water more important at low latitudes, where energy is already abundant [13], [47]. These patterns were also found in the pathogen data. In high latitude areas, mean annual temperature was the strongest climatic predictor of pathogen number and the prevalence index, whereas in tropical areas mean annual precipitation was the key climate variable. Surprisingly, population density was also a much stronger predictor in high latitude areas; one plausible reason for this finding is that there are more alternate animal hosts in the tropics, so that zoonotic pathogens may remain endemic there even when people are at low density.

## Historical data: Advantages and limitations

Cross-national differences in pathogen prevalence today have been shown to reflect differential access to disease prevention measures more than environmental variables, although pathogen richness still shows the latitudinal gradient found with other taxa [3]. Historical data on remote populations, as used here, reduces the influence of public health and modern medicine, allowing for a clearer picture of the way environmental variables shape geographic patterning in pathogen prevalence. A second advantage of this dataset for environmental analysis is that the data describe local conditions at a consistent scale, rather than being based on national averages. In the present study, the pathogen prevalence index and number of kinds of pathogens show similar patterns, and are strongly environmentally determined.

Use of historical data has limitations as well as advantages, due primarily to lack of precision in the historical source material: pathogen distributions were for the most part not available at the species level, and prevalence was assessed by an index based on ordinal scales for each pathogen, rather than by direct counts of infected individuals. Limitations of the source material also bias the pathogen sample toward vector-borne pathogens, which are less global than other pathogens [48] and are likely to show a stronger environmental signature. For this reason, prevalence and richness are more likely to be correlated with each other in this group of pathogens, even in modern datasets [49], and latitudinal gradients and climatic correlates are likely to reflect vector as well as pathogen ecology [50].

The results of this study show strong support for several theoretical and empirical findings in geographical ecology, and show that they explain human pathogen distributions on a global level. The results offer insights into past and present patterns of infectious disease, and provide information relevant to the likely effects of global warming on pathogens sensitive to temperature, frost, and seasonal dry extremes.

## Supporting Information

Datafile S1 **New Pathogen Codes for the Standard Cross-Cultural Sample: Codes and Guidance.**
(PDF)

Datafile S2 **New Pathogen Codes for the Standard Cross-Cultural Sample: Data.**
(CSV)

## References

[1] McNeill WH (1976) Plagues and Peoples. Garden City, NY: Anchor Press.

[2] Crosby AW (1986) Ecological Imperialism: The Biological Expansion of Europe, 900–1900. Cambridge University Press.

[3] Dunn RR, Davies TJ, Harris NC, Gavin MC (2010) Global drivers of human pathogen richness and prevalence. Proceedings of the Royal Society B: Biological Sciences 277: 2587–2595. 20392728 10.1098/rspb.2010.0340 PMC2982038

[4] Guernier V, Hochberg ME, Guégan JF (2004) Ecology drives the worldwide distribution of human diseases. PLoS Biology 2: e141. 15208708 10.1371/journal.pbio.0020141 PMC423130

[5] Kamiya T, ODwyer K, Nakagawa S, Poulin R (2014) Host diversity drives parasite diversity: meta-analytical insights into patterns and causal mechanisms. Ecography 37: 689–697.

[6] Robar N, Burness G, Murray DL (2010) Tropics, trophics and taxonomy: the determinants of parasite-associated host mortality. Oikos 119: 1273–1280.

[7] Kamiya T, O'Dwyer K, Nakagawa S, Poulin R (2014) What determines species richness of parasitic organisms? A meta-analysis across animal, plant and fungal hosts. Biological Reviews 89: 123–134. 23782597 10.1111/brv.12046

[8] Lindenfors P, Nunn CL, Jones KE, Cunningham AA, Sechrest W, et al (2007) Parasite species richness in carnivores: Effects of host body mass, latitude, geographical range and population density. Global Ecology and Biogeography 16: 496–509.

[9] Poulin R (2014) Parasite biodiversity revisited: frontiers and constraints. International journal for parasitology 44: 581–589. 24607559 10.1016/j.ijpara.2014.02.003

[10] Rosenzweig ML (1995) Species Diversity in Space and Time. Cambridge University Press.

[11] Willig MR, Kaufman DM, Stevens RD (2003) Latitudinal gradients of biodiversity: Pattern, process, scale, and synthesis. Annual Review of Ecology, Evolution, and Systematics 34: 273–309.

[12] Clarke A, Gaston KJ (2006) Climate, energy and diversity. Proceedings of the Royal Society B: Biological Sciences 273: 2257–2266. 16928626 10.1098/rspb.2006.3545 PMC1636092

[13] Hawkins BA, Field R, Cornell HV, Currie DJ, Guégan JF, et al (2003) Energy, water, and broad-scale geographic patterns of species richness. Ecology 84: 3105–3117.

[14] Kerr JT, Packer L (1997) Habitat heterogeneity as a determinant of mammal species richness in high-energy regions. Nature 385: 252–254.

[15] MacArthur RH, Wilson EO (1967) The Theory of Island Biogeography. Princeton University Press.

[16] Lomolino MV (2000) A call for a new paradigm of island biogeography. Global Ecology and Biogeography 9: 1–6.

[17] Whittaker RJ, Triantis KA, Ladle RJ (2008) A general dynamic theory of oceanic island biogeography. Journal of Biogeography 35: 977–994.

[18] Nunn CL, Altizer S, Jones KE, Sechrest W (2003) Comparative tests of parasite species richness in primates. The American Naturalist 162: 597–614. 10.1086/378721 14618538

[19] Barrett R, Kuzawa CW, McDade T, Armelagos GJ (1998) Emerging and re-emerging infectious diseases: The third epidemiologic transition. Annual Review of Anthropology: 247–271.

[20] Eshed V, Gopher A, Pinhasi R, Hershkovitz I (2010) Paleopathology and the origin of agriculture in the Levant. American Journal of Physical Anthropology 143: 121–133. 20564538 10.1002/ajpa.21301

[21] Cohen MN, Armelagos GJ (1984) Paleopathology at the Origins of Agriculture. Academic Press.

[22] Murdock G, White D (1969) Standard cross-cultural sample. Ethnology 8: 329–369.

[23] Cashdan E, Steele M (2013) Pathogen prevalence, group bias, and collectivism in the standard cross-cultural sample. Human Nature 24: 59–75. 23389437 10.1007/s12110-012-9159-3

[24] Murray D, Schaller M (2010) Historical prevalence of infectious diseases within 230 geopolitical regions: A tool for investigating origins of culture. Journal of Cross-Cultural Psychology 41: 99–108.

[25] Rodenwaldt E, Bader RE (1961) World-Atlas of Epidemic Diseases (1952–1961). Hamburg, Germany: Falk-Verlag.

[26] Simmons JS, Whayne TF, Anderson GW, Horack HM (1944) Global Epidemiology: A Geography of Disease and Sanitation. Philadelphia: J. B. Lippincott.

[27] Faust E, Russell P (1964) Craig and Faust's Clinical Parasitology. Philadelphia: Lea and Febiger.

[28] Low B (1990) Marriage systems and pathogen stress in human societies. American Zoologist 30: 325–340.

[29] Low BS (1994) Pathogen intensity cross-culturally. World Cultures 8: 24–34.

[30] Whiting JWM (1989) Climate data from weather stations. World Cultures 1: 179–199.

[31] Cashdan E (2003) Ethnic diversity, habitat diversity and rainfall codes. World Cultures 13.

[32] Cashdan E (2001) Ethnic diversity and its environmental determinants: Effects of climate, pathogens, and habitat diversity. American Anthropologist 103: 968–991.

[33] Eyre SR (1968) Vegetation and Soils: A World Picture. E. Arnold Ltd., second edition.

[34] Murdock GP, Wilson SF (1972) Settlement patterns and community organization: Cross cultural codes 3. Ethnology 11: 254–295.

[35] Murdock GP, Morrow DO (1970) Subsistence economy and supportive practices: Cross-cultural codes 1. Ethnology 9: 302–330.

[36] Stevens GC (1989) The latitudinal gradient in geographical range: How so many species coexist in the tropics. American Naturalist 133: 240–256.

[37] Lomolino MV (2000) Ecology's most general, yet protean pattern: The species-area relationship. Journal of Biogeography 27: 17–26.

[38] Lomolino M, Weiser M (2001) Towards a more general species-area relationship: Diversity on all islands, great and small. Journal of Biogeography 28: 431–445.

[39] Curtin PD (1989) Death by Migration: Europe's Encounter with the Tropical World in the Nineteenth Century. Cambridge University Press.

[40] Spurgin LG, Illera JC, Padilla DP, Richardson DS (2012) Biogeographical patterns and co-occurrence of pathogenic infection across island populations of Berthelots pipit (*Anthus berthelotii*). Oecologia 168: 691–701. 21983713 10.1007/s00442-011-2149-z

[41] Black FL (1975) Infectious diseases in primitive societies. Science 187: 515–518. 163483 10.1126/science.163483

[42] Bordes F, Guégan JF, Morand S (2011) Microparasite species richness in rodents is higher at lower latitudes and is associated with reduced litter size. Oikos 120: 1889–1896.

[43] Patz JA, Epstein PR, Burke TA, Balbus JM (1996) Global climate change and emerging infectious diseases. JAMA 275: 217–223. 8604175

[44] Mordecai EA, Paaijmans KP, Johnson LR, Balzer C, Ben-Horin T, et al (2013) Optimal temperature for malaria transmission is dramatically lower than previously predicted. Ecology Letters 16: 22–30. 23050931 10.1111/ele.12015

[45] Acuna-Soto R, Stahle D, Villanueva Diaz J, Therrell M (2007) Association of drought with typhus epidemics in Central Mexico. In: AGU Spring Meeting Abstracts. volume 1.

[46] Thompson RA, de Oliveira Lima JW, Maguire JH, Braud DH, Scholl DT (2002) Climatic and demographic determinants of American visceral leishmaniasis in northeastern Brazil using remote sensing technology for environmental categorization of rain and region influences on leishmaniasis. The American Journal of Tropical Medicine and Hygiene 67: 648–655. 12518857 10.4269/ajtmh.2002.67.648

[47] Whittaker RJ, Nogués-Bravo D, Araújo MB (2007) Geographical gradients of species richness: A test of the water-energy conjecture of Hawkins *et al.* (2003) using European data for five taxa. Global Ecology and Biogeography 16: 76–89.

[48] Smith KF, Sax DF, Gaines SD, Guernier V, Guégan JF (2007) Globalization of human infectious disease. Ecology 88: 1903–1910. 17824419 10.1890/06-1052.1

[49] Fincher C, Thornhill R (2008) Assortative sociality, limited dispersal, infectious disease and the genesis of the global pattern of religion diversity. Proceedings of the Royal Society B: Biological Sciences 275: 2587–2594. 18664438 10.1098/rspb.2008.0688 PMC2605802

[50] Nunn CL, Altizer SM, Sechrest W, Cunningham AA (2005) Latitudinal gradients of parasite species richness in primates. Diversity and Distributions 11: 249–256.

