# Supplementary material for: Biogeography of Human Infectious Diseases: A Global Historical Analysis
Source: PLoS One. 2014 Oct 1;9(10):e106752. doi: 10.1371/journal.pone.0106752 (PMC4182673; doi:10.1371/journal.pone.0106752)
Supplement: Datafile S1 — New Pathogen Codes for the Standard Cross-Cultural Sample: Codes and Guidance. (PDF) [file pone.0106752.s001.pdf]

New Pathogen Codes for the Standard Cross-Cultural Sample

Elizabeth Cashdan * Matthew Steele Damian Murray †

The pathogen codes described here were initially developed to test hypotheses relating variation in pathogen risk to cultural differences in collectivism [1] and authoritarianism [8] using the Standard Cross-Cultural Sample (SCCS). This document contains the code followed by information to guide its use and interpretation. The associated datafile S2 contains the coded data.

The pathogen codes

The variables in datafile S2 are as follows:

1. SCCS number
2. dengue (1=absent, 2=rare, 3=moderate/sporadic, 4=heavy/endemic)
3. filariae (1=absent, 2=rare, 3=moderate/sporadic, 4=heavy/endemic)
4. malaria (1=absent, 2=rare, 3=moderate/sporadic, 4=heavy/endemic)
5. typhus (1=absent, 2=rare, 3=moderate/sporadic, 4=heavy/endemic)
6. trypanosomes (1=absent, 2=rare, 3=moderate/sporadic, 4=heavy/endemic)
7. plague (1=absent, 2=rare, 3=moderate/sporadic, 4=heavy/endemic)
8. leishmanias (1=absent, 2=rare, 3=moderate/sporadic, 4=heavy/endemic)
9. schistosomes (1=absent, 2=rare, 3=moderate/sporadic, 4=heavy/endemic)
10. pathogen sum (sum of variables 2-9)
11. eight-pathogen z-score (mean of z-scores for 2-9, using finer scale for leishmanias and schistosomes, see text)
12. pathogen prevalence index (a ten-pathogen z-score, adding spirochetes and leprosy, from Low's code)
13. pathogen richness (number of pathogens present, of 10)

The eight columns to the right of the SCCS ID number indicate the pathogen prevalence on an ordinal scale (1–4) for each pathogen, and column 10 ("pathogen sum") is the sum of those values. It can be used as a good summary index of pathogen prevalence. However, we also wanted to take advantage of additional data where available. This required normalization of different scales, and produced the following two additional indices.

*Department of Anthropology, University of Utah, cashdan@anthro.utah.edu
†Department of Psychology, UCLA

8-pathogen z-score For most pathogens we had only aggregate data on related species, but we had separate data for the three species of schistosomes and for two groups of leishmanias (visceral and cutaneous/mucocutaneous leishmanias). In order to take advantage of the finer-grained data we summed the prevalence levels across the three schistosome types (possible values 3–12) and both leishmania types (possible values 2–8) and converted these and the values for the other pathogens into z-scores. The average of these z-scores is the variable called "8-pathogen z-score".

pathogen prevalence index The pathogen prevalence index takes advantage of two additional pathogens—spirochetes and leprosy—that were coded by Low [4, 5] using different sources. We converted those data from their original 3-point scale to z-scores, and averaged them with the z-scores used in the 8-pathogen z-score. The resulting variable is the pathogen prevalence index used in our analyses.

Pathogen richness In order to get a measure that more closely reflects species richness, we dichotomized each pathogen as either present or absent, and summed those values. That score ("pathogen richness") is the number of pathogens present out of a total possible of 10.

Methods

Sample and sources. The Standard Cross-Cultural Sample (SCCS) of 186 non-industrial cultures is pinpointed by date and geographic coordinates to a key ethnographic description of each society [6]. We used three sources of historical pathogen data in order to match these coordinates as closely as possible. Our main sources were the three-volume series of maps in Rodenwaldt and Bader [9] and the maps and data in Simmons et al. [10]. For two pathogens we also referred to information in Faust and Russell [3]. The geographic resolution of the historical maps allowed us to assess pathogen prevalence at the target location, but the temporal resolution was less precise. The pathogen data describe conditions prevailing in the early-mid twentieth century, slightly later than the target dates of most of the SCCS societies (interquartile range = 1880-1939), and significantly later than a few historical societies also included in the SCCS. Omitting those societies did not change the results in this paper, so they were left in the final sample.

Procedure. We coded the prevalence of eight pathogens: leishmanias, trypanosomes, malaria, schistosomes, filariae, dengue, typhus, and plague. The choice of pathogens was determined chiefly by whether or not our sources contained adequate global coverage. We excluded pathogens where coverage was only partial in our sources (e.g., leprosy), where recent distributions had changed markedly (e.g., smallpox), and where the effects were not seriously debilitating or life-threatening (e.g., hookworm). Methods for coding were adapted from Murray and Schaller [7], who used the same sources and methods in coding historical disease prevalence within modern geopolitical regions. Due to the relative antiquity of the source materials and issues with precisely assessing levels of historical disease prevalence, our coding scheme was relatively crude. A 4-point coding scheme was employed: 1 = absent, 2 = rare, 3 = sporadic or moderate prevalence, 4 = present at severe levels or epidemic. These values were for the most part taken directly from isolines on the maps indicating prevalence levels, but in a few cases where map data were inadequate we also used verbal descriptions found in Simmons et al. [10].

The highest value within a circle of 100 km radius around each culture's target location determined the pathogen value for the culture. An exception was made in 6 cases (4 for dengue and 2 for schistosomes) where a city within the 100 km area had a high level not found in the surrounding region that contained the SCCS culture. In thoses cases the highest value in the surrounding region was used.

Intercoder Reliability. In order to ensure consistency of coding criteria between the current disease prevalence measure and previous measures using these sources, two coders, one of whom was

primarily responsible for creating the historical disease index measure reported in Murray and Schaller [7], independently coded the historical prevalence of the eight pathogens for each of the 66 SCCS cultures in the Africa world region.

The coding scheme appeared to produce consistent pathogen prevalence estimates between coders. For the initial total pathogen prevalence ratings for the African cultures the interrater agreement was high, $r = .94$. In total, the two coders independently provided identical disease scores for 365 of the 528 (69%) pathogen prevalence estimates that were provided by both coders. For 158 (30%) of the pathogen prevalence estimates, the coders differed by one point. On only 5 of the 528 pathogen prevalence estimates was the disparity between the coders 2 points (0.9% of cases), and for none of the estimates was the disparity higher than two. In the rare cases that the pathogen prevalence scores different by two, the two coders examined each of the cases together in order to find any potential bias in the coding criteria. Examining these five cases revealed no evidence of systematic bias or consistent differences in the coding criteria used by each coder.

Comparison with existing codes

The prevalence index combines data on the 8 pathogens that we coded and 2 pathogens from published codes developed by Low [5]. We embarked on this project in order to get additional data, not because of any concerns about Low's codes. Using the two codes allows for an independent check on analyses, because the two codes were based on different sources and include some different pathogens. Our combined index also incorporates more data than is available in either coding alone.

Five pathogens (leishmanias, trypanosomes, malaria, schistosomes, and filariae) are in both codes, while two (spirochetes and leprosy) are only in Low's and three (dengue, typhus, and plague) are only in ours. The correlation between the two codes based on pathogen sums of just the five included in both is $r = .80$ ($n = 186, p < 0.0001$).

Choice of diseases affects geographical patterning. While the global environmental model in the paper did equally well in predicting both the pathogen prevalence index and Low's total pathogen stress index ($R^2 = .58$ for both) some interesting differences emerged due to differences in the pathogens included in each dataset. Mean annual temperature was a stronger predictor of Low's index, while population density was a stronger predictor of ours. This is probably because Low's index does not include typhus and plague—two of only three pathogens whose prevalence was unrelated to temperature in our data. Any two collections of pathogens will differ somewhat in biogeographical patterning, and we discuss these biases below.

Both codes are biased toward pathogens transmitted by insect and other vectors, some of which also have non-human hosts. The bias in our data arose from limitations in the source material, and has both advantages and disadvantages. The most obvious disadvantage is that some important diseases spread via droplet and oral-fecal transmission, such as measles and cholera, are omitted in both codes. The associated advantage is that pathogens transmitted directly from person to person rather than via vectors can spread more rapidly across the globe by international travel. They are probably also more easily controlled by modern health-care measures than are pathogens that remain endemic in other species. The data are biased, therefore, towards pathogens least likely to have undergone recent rapid changes in geographic distribution, which makes them less sensitive to mismatches in time with the SCCS target dates, and more stable for cross-cultural comparisons.

The pathogen prevalence index reflects environmental variables more strongly than pathogen prevalence data used in modern cross-national data [2]. This may reflect its bias toward vector-transmitted pathogens, but it probably also reflects the fact that the SCCS data are localized to remote areas and were collected at a time when disease distributions were less affected by public health and modern medicine than is the case today.

References

[1] Cashdan, E. and Steele, M. (2013). Pathogen prevalence, group bias, and collectivism in the standard cross-cultural sample. *Human Nature*, 24(1):59–75.

[2] Dunn, R. R., Davies, T. J., Harris, N. C., and Gavin, M. C. (2010). Global drivers of human pathogen richness and prevalence. *Proceedings of the Royal Society B: Biological Sciences*, 277(1694):2587–2595.

[3] Faust, E. and Russell, P. (1964). *Craig and Faust's Clinical Parasitology*. Lea and Febiger, Philadelphia.

[4] Low, B. (1990). Marriage systems and pathogen stress in human societies. *American Zoologist*, 30(2):325–340.

[5] Low, B. S. (1994). Pathogen intensity cross-culturally. *World Cultures*, 8(1):24–34.

[6] Murdock, G. and White, D. (1969). Standard cross-cultural sample. *Ethnology*, 8(4):329–369. Updated at `http://www.escholarship.org/uc/item/62c5c02n`.

[7] Murray, D. and Schaller, M. (2010). Historical prevalence of infectious diseases within 230 geopolitical regions: A tool for investigating origins of culture. *Journal of Cross-Cultural Psychology*, 41(1):99–108.

[8] Murray, D. R., Schaller, M., and Suedfeld, P. (2013). Pathogens and politics: Further evidence that parasite prevalence predicts authoritarianism. *PloS One*, 8(5):e62275.

[9] Rodenwaldt, E. and Bader, R. E. (1961). *World-Atlas of Epidemic Diseases (1952-1961)*. Falk-Verlag, Hamburg, Germany.

[10] Simmons, J. S., Whayne, T. F., Anderson, G. W., and Horack, H. M. (1944). *Global Epidemiology: A Geography of Disease and Sanitation*. J. B. Lippincott, Philadelphia.
